# Self-Centering Shape Memory Alloy-Viscoelastic Hybrid Braces for Seismic Resilience

**DOI:** 10.3390/ma15072349

**Published:** 2022-03-22

**Authors:** Zhe-Xi Zhang, Yiwei Ping, Xiuzhang He

**Affiliations:** 1 Department of Structural Engineering, College of Civil Engineering, Tongji University, Shanghai 200092, China; zzx888@tongji.edu.cn (Z.-X.Z.); xiuzhanghe@gmail.com (X.H.); 2 Department of Building and Real Estate, The Hong Kong Polytechnic University, Hong Kong, China

**Keywords:** self-centering, shape memory alloy (SMA), viscoelastic, brace, hybrid control, seismic resilience

## Abstract

This paper presents a novel type of hybrid self-centering braces incorporating tension-only superelastic NiTi shape memory alloy (SMA) cables and integrated viscoelastic dampers (VEDs). One of our reasons for proposing this new SMA-viscoelastic hybrid brace (SCVEB) is to provide enhanced energy-dissipation ability whilst promoting increased self-centering tendency compared with the existing SMA-based self-centering solutions, where upgrading behavior is mainly benefited from the participation of the VEDs. The configuration and the working principle, along with theoretical equations describing the mechanical behavior of the SCVEB, are described in detail firstly. Experimental verification of individual elements in this SCVEB system, namely the NiTi SMA cables and VEDs, was performed to obtain a basic understanding of their mechanical properties. A proof-of-concept SCVEB specimen was then manufactured, and its cyclic performance was further investigated. Followed by this, a system-level analysis on a series of steel frames equipped with or without SCVEB was conducted. The results showed that the SCVEB system exhibited a moderate damping ratio and a more efficient controlled behavior in terms of its post-event residual deformation and floor acceleration when compared with those of the non-SCVEB system.

## 1. Introduction

The past decades have witnessed several major earthquakes [1,2,3,4], and the structures designed by modern ductility-based seismic design philosophy are proven to be effective in providing sufficient life safety assurance during the earthquakes. However, this design philosophy may be no longer sufficient when special attention is given to the issue of the economic seismic loss. For example, the 2011 Christchurch earthquake caused damage to thousands of buildings that did not collapse but were unrecoverable due to unacceptable damage and residual deformation, requiring a heavy cost to repair them [4]. It has been suggested that the magnitude of residual inter-story drift should be controlled within a drift limit of 0.5%, beyond which the structures may no longer be economically feasible to repair from the perspectives of building functionality, construction tolerances, and safety [5]. 

Driven by this demand, the community of seismic engineers has made continuous efforts to improve the post-earthquake structural performance over the past years. A promising solution, which is known as self-centering framed structural system, has received extensive research interests [6,7]. Additional self-centering members/devices, such as beam-to-column connections [8,9,10,11,12,13,14,15,16,17], braces [18,19,20,21,22,23,24,25,26,27], and dampers [28,29,30,31,32,33,34,35,36,37,38,39,40,41], with the capability of returning to their initial resting position after earthquake, are proven to be effective in eliminating the residual story drifts when they are installed in the corresponding frame systems. Ricles et al. [42,43] developed a post-tensioned (PT) moment-resisting connection where seat angles were utilized as an energy-dissipation source. Under loading, gap-opening occurred at the beam–column interface and the PT tendons provided a restoring force by acting to close the gap. Maurya and Eatherton [44] developed a new self-centering beam for moment frame that eliminated deformation incompatibility with the gravity framing. In the case of braces, Christopoulos et al. [18] proposed a new bracing system that comprised of tensioning elements and friction pads. Chou et al. [6,27] experimentally examined a dual-core self-centering sandwiched buckling restrained brace to increase deformability capacity. However, there still exists some inevitable issue associated with the PT self-centering technology. For example, due to the limited elastic strain (about 2%) of the PT tendons [18,27], there may be a deformation capacity deficiency, which may put the self-centering framed structures at risk when a severe earthquake happens. Without a redundant load-carrying path, yielding or/even rupture of the PT tendons would lead to serious disaster, and the lateral load resistance may be totally lost, which may trigger the collapse of structure. On the other hand, the cyclic response of the PT self-centering system generally exhibits a typical “flag-shape” hysteretic behavior, whose energy-dissipation capability (can be readily understood as the area enclosed in the hysteretic loops) is significantly reduced compared to that of conventional structural systems. The reduced energy dissipation may lead to the amplification of peak-deformation response and floor acceleration [45,46]. It has been recognized that the magnified peak response would cause extensive damage to both structural and non-structural components, which should be addressed in the practical design. 

In recent years, various improvement strategies have been proposed to address the issues associated with PT self-centering system mentioned above. For instance, shape memory alloys (SMAs) [47,48,49,50], especially superelastic NiTinol SMA, were introduced in self-centering systems [51,52,53,54,55] because of their good energy-dissipation ability, ductility, and fatigue resistance. Superelasticity refers to the capability to spontaneously recover when the load is removed from SMA elements after experiencing a large strain up to 8~10% [56,57,58]. Evidently, SMA-based tendon provides a very competitive deformation compared with that of the aforementioned conventional PT tendons. Miller et al. [59] investigated a self-centering buckling-restrained brace (SC-BRB) by using SMA rods as a source of restoring force, which successfully achieved appreciable energy dissipation, large deformation capacity, and self-centering ability. Zhu and Zhang [38] developed a self-centering friction damping brace (SFDB). The study showed that the frame equipped with SFDB was capable of achieving a seismic-response level that is comparable with that of BRBF, but with a significant reduction in residual drifts. Chen et al. [60] employed variable friction devices to improve energy-dissipation efficiency of a brace without compromising the self-centering ability of pre-tensioned SMA cables.

With initial confidence gained from these pioneering investigations, a concept of SMA-viscoelastic hybrid braces (SCVEB) is proposed in this paper. The motivation behind this concept is to move a further step to deal with the issues arising from a self-centering system in previous studies, such as the amplified peak deformation and floor acceleration. The concept has been preliminarily examined by the authors and co-workers through a numerical study [61]. In this paper, a proof-of-concept experiment on a prototype SCVEB device was conducted, and the influence of some key parameters on the seismic behavior was discussed. Followed by this, a system-level analysis that investigated the effectiveness of the proposed SCVEB was conducted.

## 2. Configuration and Working Principle of SCVEB

**Figure 1:**
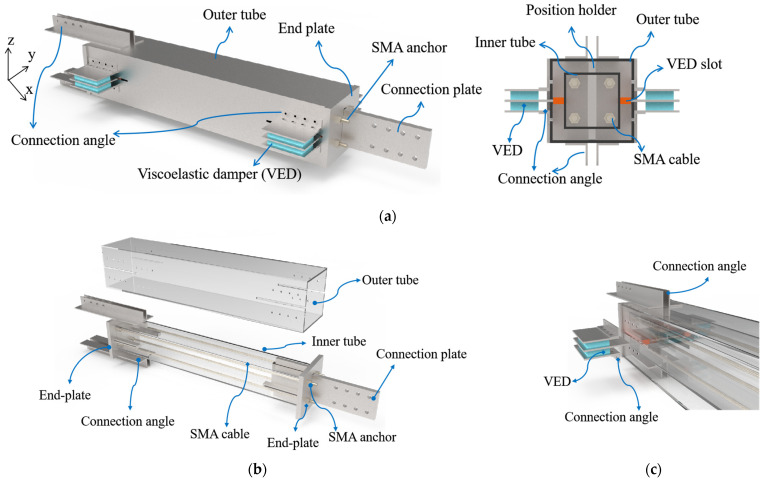
Three-dimensional schematic illustration of (**a**) SMA-viscoelastic hybrid braces (SCVEB), as well as its (**b**) self-centering (SC) system and (**c**) energy-dissipation (ED) system.

The configuration of the investigated SCVEB is illustrated in Figure 1. Conceptually, the proposed SCVEB can be decomposed into two systems, i.e., self-centering system (SC system) and energy-dissipation system (ED system). The former is mainly functioned by the SMA elements, and the latter mainly relies on the energy-dissipation property of viscoelastic materials [62,63,64]. Note that SMA elements also consume some of the input energy when an earthquake occurs. In this section, some basic concepts, as well as the configuration of the SCVEB, are introduced, and the associated analytical expression for the behavior of the SCVEB is presented.

### 2.1. SC System

The studied SCVEB employs SMA cables [65,66] as the core part of the SC system, as shown in Figure 1b. It is worth mentioning that various types of SMA elements (such as the disc spring [67], ring spring [57,68,69,70,71], washer spring [72], etc.) have been developed and are welcomed to be incorporated in this SCVEB system with some necessary modifications on the configuration. Other necessary components include the outer tube, inner tube, end-plates, angles, connection plates, position holders, and tightening nuts. The end-plates are not connected to either the outer or inner tubes; therefore, they can slide along the brace length freely when SCVEB works. On one side of the brace, a connection plate passes through the slot cut in the end-plate and is welded to the inner tube, serving as the connecting component to the structural systems. On the other side, two pairs of double angles are welded to the outer tube to accommodate the connection.

During assembly, the SMA cables are firstly housed in the inner tube. Then the inner tube is inserted inside the outer tube concentrically and positioned with two position holders placed between them. SMA cables are connected to the end-plates via a threaded junction. The end-plates are subsequently placed on both side of the outer tube. Finally, the SMA cables are pre-tensioned to obtain sufficient initial stiffness and desired “yield” resistance. It is believed that an appropriate pre-tension level can encourage the SMA cables to reach their full potential of self-centering property [60,73]. The possible method to apply the pre-tension force is explained later.

**Figure 2:**
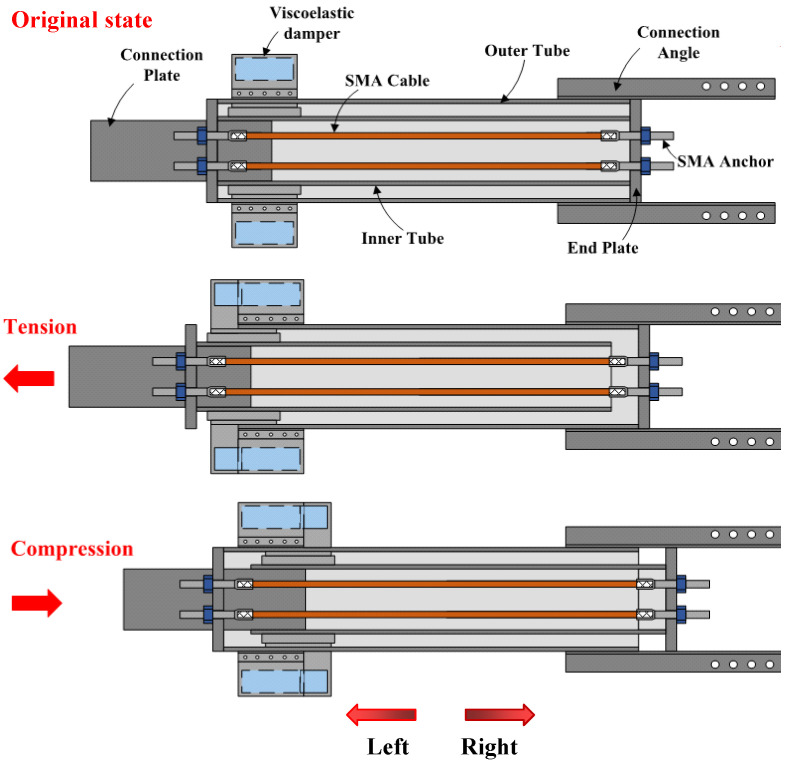
Working principle of SCVEB.

**Figure 3:**
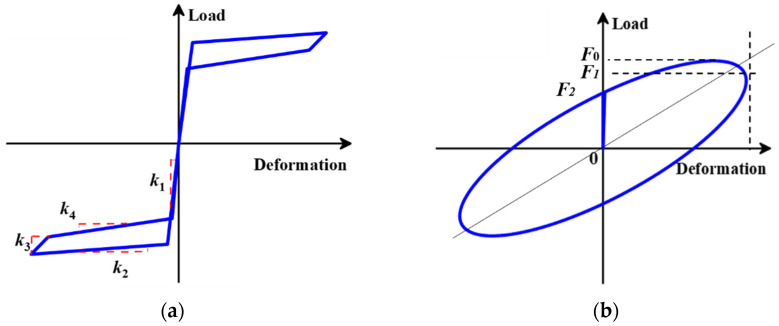
Illustration of hysteretic curves: (**a**) hysteresis model of SMA-based elements/components and (**b**) Kelvin–Voight model for viscoelastic materials.

The working principle of the SC system is further demonstrated in Figure 2. For ease of description, we assume that the outer tube is completely fixed and the inner tube is driven by the connection plate. The terms “tension” and “compression” refer to leftward or rightward movement direction of the inner tube relative to the outer tube, respectively, as the orientation marked in Figure 2. When the brace is in tension, the left end-plate is pushed away from the outer tube by the inner tube, while the right end-plate is blocked by the outer tube. Similarly, when the brace is in compression, the inner tube, together with the connection plate, moves rightward and pushes the right end-plate away from the outer tube, while the left end-plate is blocked by the outer tube. Thus, both compression and tension brace deformations cause the two end-plates to move apart, elongating the SMA cables and increasing their tension force concurrently. Therefore, the SMA cables always remain in tension no matter what condition state the brace is in, giving full play to the performance of the SMA cables. Previous studies have verified that the SMA-based elements/components are expected to exhibit a flag-shaped response [67,74,75,76]. An idealized/simplified force-displacement relationship is adapted here to capture this unique hysteresis evolutionary path (see Figure 3a), where four stiffness parameters, namely *k*_1_ for loading stage, *k*_2_ for loading plateau stage, *k*_3_ for unloading stage, and *k*_4_ for unloading plateau stage, are considered [77]. This four-segment simplified model was further cooperated in OpenSEES [78] software for the system-level analysis.

### 2.2. ED System

Extra energy dissipation of the SCVEB is provided by the viscoelastic dampers (VEDs). The participation of the viscoelastic material is also expected to provide certain stiffness for the brace especially after the SMA-based elements advance into the post-yield plateau stage. As shown in Figure 1c, the ED system (i.e., the VED) consists of three plates where two rubber layers are sandwiched between them. The two external plates are bolted to the outer tube by steel angles, and the middle plate is inserted into the slot on the inner tube. Slot cuts are made on the outer tube to allow the middle plate to pass through and move freely when the inner and outer tube move apart. 

When the ED system functions, viscoelastic materials would act via shear deformation between two steel plates. It is worth noting that the ingredient (e.g., resin content) of viscoelastic materials may strongly affect their linear/nonlinear characteristic; thus, their hysteresis models vary. The so-called linear characteristic refers to the ellipse-shaped hysteresis curve, which is featured by most typical viscoelastic materials (see Figure 3b). Kelvin–Voight model associated with linear viscoelastic materials was adopted herein to derive the theoretical formulas of the proposed SCVEB, since this model is most widely accepted for the viscoelastic materials.

The restoring force, *F*_ve_, can be expressed as follows:

(1) $$F_{\mathrm{ve}}=K_{\mathrm{eff}}\cdot u+C_{e}\cdot v^{\alpha }$$

where *u* is the displacement, *v* is the velocity, *α* is the velocity exponent, and *K*_eff_ is the equivalent storage stiffness, which is determined by the following:

(2) $$K_{\mathrm{eff}}=\frac{nG^{\prime }A}{h}$$

where *n* is the number of viscoelastic material layers, *A* is viscoelastic material’s shear section area, and *h* is the thickness of a single viscoelastic layer; the storage modulus, *G*’, is determined by the following:

(3) $$G^{\prime }=\frac{F_{1}h}{nAu_{0}}$$

where *u*_0_ is the maximum displacement during loading (as illustrated in Figure 3b), and *F*_1_ is the damping force corresponding to *u*_0_.

*C*_e_ is the equivalent damping coefficient, which is calculated by the loss modulus, *G*″, and the circular frequency, *ω*:

(4) $$C_{e}=\frac{nG^{\prime \prime }A}{\omega h}$$

The loss modulus, *G*″, can be determined by the following:

(5) $$G^{\prime \prime }=\frac{F_{2}}{F_{1}}G^{\prime }$$

where *F*_2_ is the damping force corresponding to the displacement at zero.

### 2.3. Theoretical Activation Force of SCVEB

An activation (or sometimes called “decompression”) force, *F*_a_, which should be reached firstly before the SMA cables enter the working situation, can be expressed by the following:

(6) $$F_{a}=\frac{F_{a}}{k_{\mathrm{br}}}\cdot k_{2}+F_{p}$$

where *F*_p_ is the total cable preload; *k*_2_ is the “post-yield” stiffness, i.e., that of the forward transformation plateau, as marked in Figure 3a; and *k*_br_ is the initial stiffness of the brace prior to activation:

(7) $$k_{\mathrm{br}}=k_{\mathrm{out}}+k_{\mathrm{in}}+k_{2}$$

where *k*_in_ and *k*_out_ are the elastic axial stiffness of the inner and outer tubes, respectively. Since *k*_br_ >> *k*_2_ for most cases, Equation (6) can be subsequently reduced to the following:

(8) $$F_{a}\approx F_{p}$$

Note that there is no relative deformation before activation; therefore, the load resistance provided by VED, i.e., *F*_ve_, can be considered as zero. However, when the brace is subjected to dynamic excitations, the velocity-related term in Equation (1) should not be ignored. In this case, the total activation force of SCVEB is as follows:

(9) $$F_{a}=F_{p}+C_{e}\cdot v^{\alpha }$$

**Figure 4:**
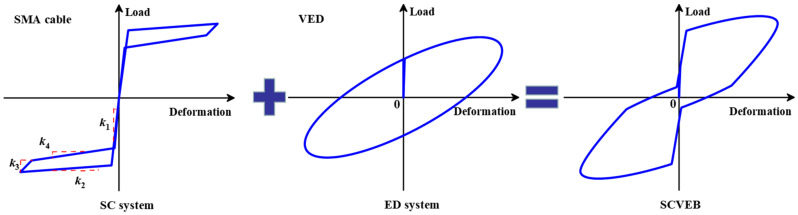
Typical hysteretic behavior of SCVEB.

Figure 4 shows the theoretical load–deformation relationship of the proposed SCVEB. Ideally, after activation, SCVEB’s load at any deformation can be described as the sum of the forces provided by the above two systems.

## 3. Investigation on Kernel Elements 

### 3.1. Individual SMA Cable Test

**Figure 5:**
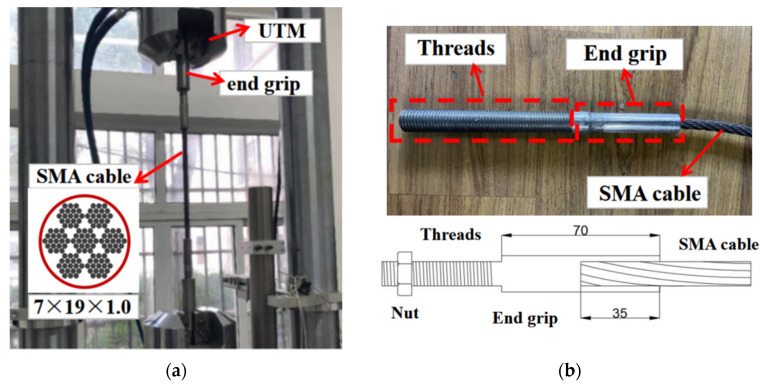
Information of SMA cable: (**a**) test setup and basic construction; (**b**) end grips.

Figure 5a shows the geometric configuration of a typical SMA (50.8 at.% nickel–49.2 at.% titanium alloys) cable that was later adopted in the SCVEB. The SMA cable consists of seven helically wrapped strands, each of which contains 19 helically wrapped monofilament SMA wires with a diameter of 1.0 mm (designated as 7 × 19 × 1.0). The effective length of the studied SMA cable is about 300 mm, and special end grips were machined for the SMA cables. The cable segment was first cut from a long cable by local melting. The hot ends of the SMA cable were inserted into the preprocessed hole of the end grips, followed by a machinal squeezing process. As a result, the cable ends were housed in the squeezed end grips tightly. The end grips were further machined to be threaded for connection and pre-tensioning, as shown in Figure 5b.

**Figure 6:**
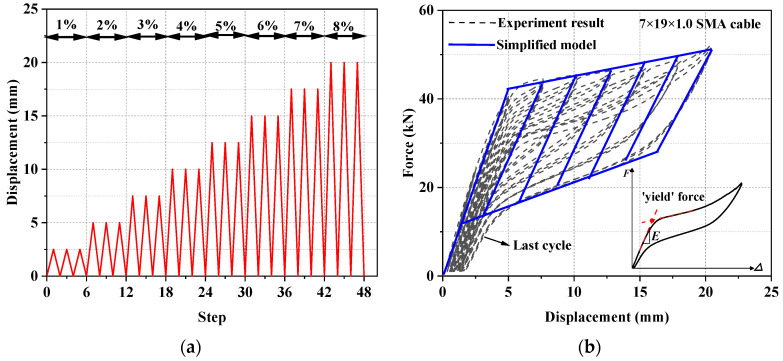
Test of SMA cable specimen: (**a**) loading protocol and (**b**) test results.

Pseudo-static tension testing of the SMA cable was conducted on a Universal Test Machine (UTM) to obtain their basic mechanical behavior. The displacement and the applied load were monitored by the grip displacement and the built-in load cell in the UTM. A displacement-controlled incremental loading protocol was applied based on a displacement interval *Δ*_1_ of 2.5 mm, which is equal to an axial deformation of 1% of the test cable sample. The loading began with three cycles each at *Δ*_1_, 2*Δ*_1_, 3*Δ*_1_, 4*Δ*_1_, 5*Δ*_1_, and 6*Δ*_1_, as shown in Figure 6a. 

The force–displacement curve is shown in Figure 6b. It can be seen that the studied SMA cable exhibited a satisfactory self-centering capability, and the flag-shaped hysteresis became stabilized after a few loading cycles. The residual strain was generally small. The aforementioned four segment stiffnesses, *k*_1_, *k*_2_, *k*_3_, and *k*_4_, were measured as 22,500, 1500, 18,000, and 3000 MPa, respectively. A dimensionless index, namely equivalent viscous damping (*EVD*), is employed to evaluate the energy-dissipation capacity and is expressed by the following:

(10) $$EVD=\frac{1}{4\pi }\frac{E_{D}}{E_{S}}$$

where *E_D_* is the typical energy dissipation per cycle (i.e., the area within the inelastic force-displacement response curve), and *E_S_* is the recoverable elastic strain energy stored in an equivalent linear elastic system. The calculation results confirm that the studied SMA cables have moderate energy-dissipation capability (*EVD* = 2.6% at the maximum tested displacement).

### 3.2. Viscoelastic Material Test

**Figure 7:**
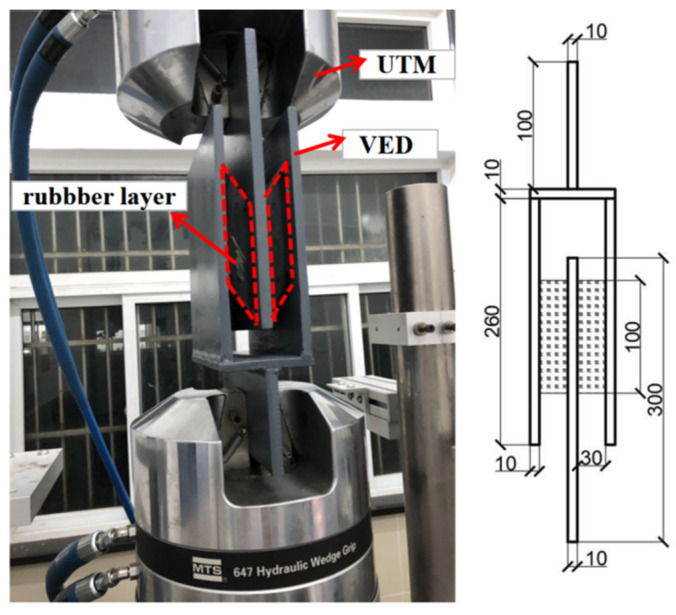
Test setup of viscoelastic material.

**Figure 8:**
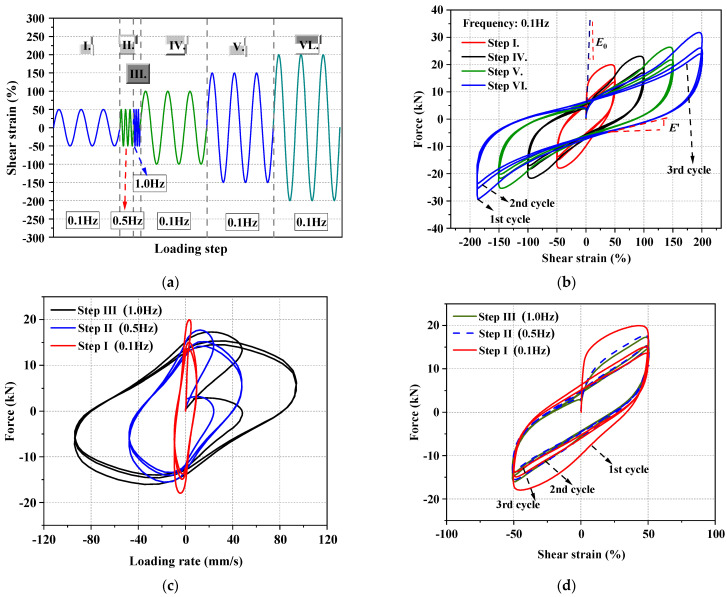
Tests of rubber layer: (**a**) loading protocol and (**b**–**d**) test results.

A preliminary test was conducted by using a UTM to investigate the damping behavior of viscoelastic material. The configuration and geometry dimensions of the test setup are shown in Figure 7. The shearing area and thickness of the studied rubber layer are 100 × 180 mm^2^ and 30 mm, respectively. To obtain a comprehensive understanding of the rubber layer under various loading scenarios (e.g., different loading amplitudes and different loading frequencies), a progressive hysteresis loading protocol with six loading steps was conducted, as drawn in Figure 8a.

The test results are plotted in Figure 8b–d. It can be seen that the first cycle of all of these hysteretic loops is plumper than that of the rest cycles, with significant initial stiffness (*E*_0_), regardless of what the loading amplitudes is. The peak strength decreases when the reverse loading is repeated under the same amplitude, whereas it increases as the loading amplitude expands, indicating that the rubber features strong nonlinear characteristics, both cyclic softening and cyclic hardening. Moreover, the rubber’s loading/unloading plateau stiffness (*E*′) decreases as incremental loading process progresses. The hysteresis force–shear strain relationship exhibits obvious loading amplitude dependence, where Mullins effect is usually accepted to explain this phenomenon [79]. The hysteretic loop of the studied rubber shows a typical non-linear characteristic [80], a phenomenon which is mainly attributed to the temperature rise, as well as the fatigue performance of this material during cyclic loading [81]. Figure 8c,d shows the relationship between the damping force and loading frequency. It can be seen that the generated peak loads at the maximum loading displacement are not sensitive to the loading frequency, and the load–deformation relationship would remain stable since the second cycle. Therefore, it is reasonable to assume that the velocity-dependent property of the rubber purchased for this study was not significant. Nonetheless, they are still commonly called “viscoelastic material” in the community of civil engineers, as they exhibited many typical behaviors of viscoelastic dampers, such as similar hysteresis, high damping capability, temperature dependence, Mullins effect, etc. A similar phenomenon was also observed in previous works [62,82]. The difference between linear and nonlinear viscoelastic materials probably lies in the energy-dissipation amount and the load under peak deformation (see Figure 3b and Figure 8b for a more clarified understanding). Note that other types of energy-dissipation devices (such as wire rope isolator [83]) also feature similar hysteretic behaviors, so it is feasible to employ those devices in the proposed hybrid braces in future work.

## 4. Experimental Verification of Proposed SCVEB

### 4.1. Information of SCVEB Specimen

**Figure 9:**
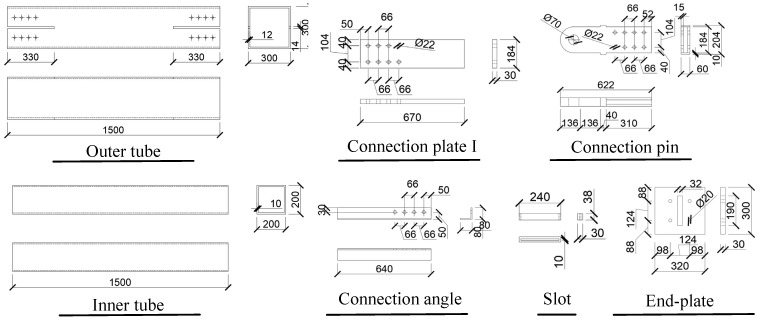
Dimensions of the SCVEB (units in mm).

A proof-of-concept SCVEB specimen was fabricated and tested. The components were fabricated in the shop and assembled in the laboratory. The corresponding configuration and assembling method for the SCVEB specimen have been described in Section 2. The key dimensions of the prototype SCVEB specimen are given in Figure 9. 

The main components (i.e., outer tube, inner tube, connecting angles, end-plates, and other necessary accessory components) were manufactured by Grade Q345 steel (nominal yield strength: 345 MPa). The rubbers were custom manufactured to the designed geometric shape and employed in the ED system of the SCVEB. Four SMA cables, whose properties were investigated in Section 3.1, were employed in the SCVEB. The length of the SMA cable was finally designed as 1770 mm. It is worth noting that, in practice, the SMA cables do not need to run the full length between the end-plates, as they have a high capacity of recoverable strain. Alternatively, high-strength steel cables may fill in for the rest of the length. Shortening the length of the SMA cable could promote an increased efficiency of the SMA cable and save the total production cost of the SCVEB at the same time. 

**Figure 10:**
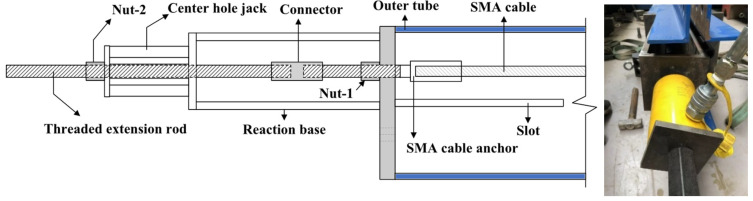
Pre-tension equipment.

The end grips of each cable were threaded, through which design the cables were finally fastened to the end-plates, as shown in Figure 5b. Prior to starting the follow-up experiments, the SMA cables were pre-tensioned through specially designed pre-tensioning equipment. As shown in Figure 10, a reaction base was required due to the limited stroke capacity of the hydraulic jack. When tensioning, the hydraulic jack was operated via a threaded extension rod that was connected to the threaded end grip by a coupler. Once the targeted pre-tensioned level (30 kN) was achieved, the nuts were locked and the pre-tensioning process finished.

### 4.2. Test Setup, Instrumentation, and Loading Protocol

**Figure 11:**
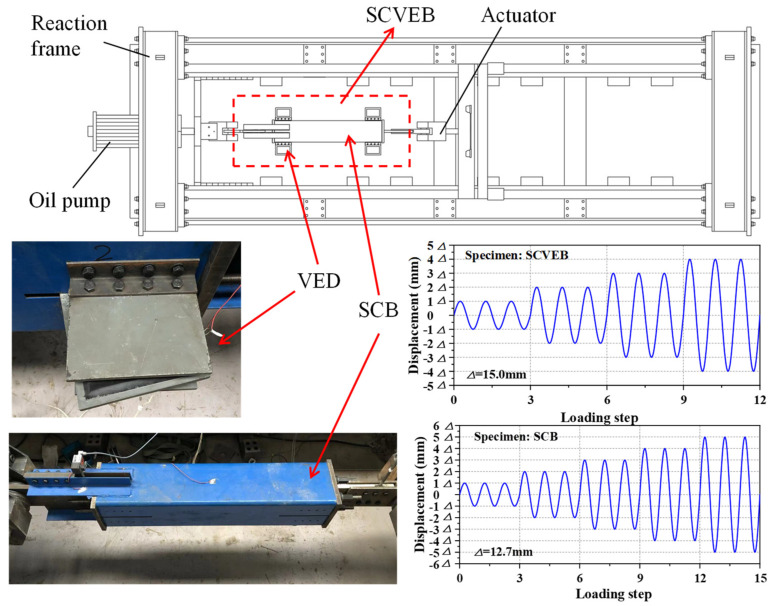
Test arrangement and observation.

The test setup for the SCVEB specimen is schematically shown in Figure 11. The whole loading plane was oriented horizontally and the cyclic load was applied axially to the SCVEB specimen. An electro-hydraulic servo actuator with a maximum loading rates of 2000 mm/s was employed to conduct this experiment. Two ends of the SCVEB were pinned to the servo actuator and strong base on the reaction frame, respectively. Through this method, the brace can be viewed as subjected to uniaxial force during cyclic loading. The deformation was measured by two vertical linear variable differential transformers (LVDTs), which were attached to the connection plate of the inner tube and the connection angle of the outer tube, respectively. A series of longitudinal strain gauges were placed on the SCVEB surface to real-time-monitor whether the steel elements stayed elastic during the whole process. The uniaxial force applied to the brace was measured by the built-in load senor of the servo actuator.

The test was conducted in two rounds. Firstly, the complete SCVEB, i.e., with SMA cables + VEDs, was tested. Then the VEDs were disassembled from the SCVEB (denoted as SCB in the following discussions) to examine the hysteretic behavior of the isolated SC system. The loading protocols for the successive component-level tests can be seen in Figure 11 [84]. For the SCVEB test, an incremental amplitude loading protocol with the sequence of 1*Δ*_v_, 2*Δ*_v_, 3*Δ*_v_, and 4*Δ*_v_, where *Δ*_v_ = 15 mm, was employed. The interval *Δ*_v_ corresponds to the deformation amount when the rubber layer experiences a shear strain of 50%, a value commonly adopted for the investigation of viscoelastic material-based damper [80,81]. Such a loading protocol was practiced twice with two loading frequency levels, i.e., 0.1 and 1.0 Hz, successively. For the SCB test, an incremental amplitude-loading protocol with loading frequency of 0.1 Hz was adopted, following the sequence of 1*Δ*_b_, 2*Δ*_b_, 3*Δ*_b_, 4*Δ*_b_, and 5*Δ*_b_, where *Δ*_b_ = 12.7 mm. The interval *Δ*_b_ corresponds to the 1% strain level of the SMA cable. All of these amplitudes were input in a sinusoidal wave format, and each amplitude was repeated for three times before moving to the next amplitude.

### 4.3. Test Results and Discussions

**Figure 12:**
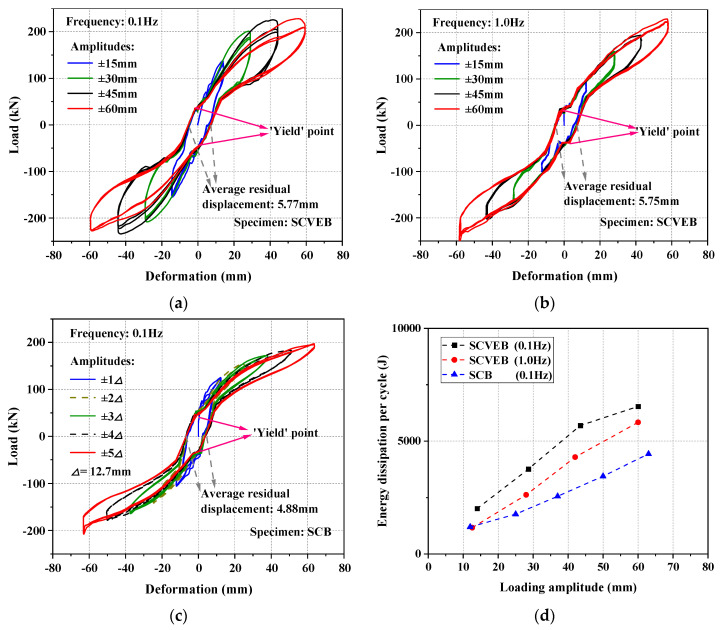

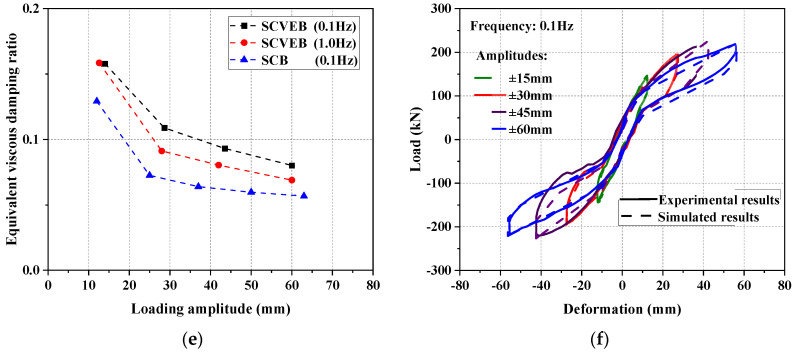
Test results for (**a**) SCVEB-0.1 Hz series, (**b**) SCVEB-1.0 Hz series, (**c**) SCB-0.1 Hz series, (**d**) energy-dissipation per cycle, (**e**) *EVD*, and (**f**) comparison between the experimental hysteresis loops and the simulated results.

The load–deformation hysteretic curves of the SCB and SCVEB specimens are plotted in Figure 12. Some important performance indicators are marked in the figure.

It can be seen that a non-linear hardening slope is followed after the elastic range. There is an obvious turning point (“yield” point) in the curve, as depicted in Figure 12a–c. The reason of this phenomenon is mainly attributed to the pre-tensioning status of the SMA cables before decompression. The “yield” loads for SCVEB and SCB are both around 30 kN, a value which is consistent with the amount of the pre-tensioned load. This indicates that the “yield” phenomenon is resulted from the decompression of the pre-tensioned SMA cables. Theoretically, the residual displacement of the SCB would be zero when the load is removed (see Figure 4). Viewing the result of 0.1 Hz series SCB test; however, the residual deformation did not return to zero as expected, but instead remained at a constant value during different loading cycles. This undesirable residual deformation may be due to defects in the brace itself (such as imperfect mechanical factors introduced during machining, manufacturing, and assembly), rather than the performance degradation of the materials (i.e., SMA cables), since SMA cables’ self-centering capability has been confirmed by the tests aforementioned in Section 3.1. In fact, the measured length of the outer tube is about 5 mm longer than that of the inner tube, which deviates from the original design that the lengths of the outer and inner tube should be the same. It should be noticed that the residual deformations of SCB and SCVEB were 4.88 mm and 5.77 mm (under 0.1 Hz), respectively; the results were very close to the aforementioned manufacturing error length between outer and inner tube (5 mm). Therefore, it can be concluded that the imperfect self-centering results were mainly resulted from the manufacturing errors. Although the manufacturing errors resulted in a maximum residual displacement of 4.88 mm in the specimen that only contains SMA cables (i.e., specimen SCB, as shown in Figure 12c), it did not much affect the completed brace specimen (i.e., SCVEB) to achieve the predicted hysteretic response, as shown in Figure 4. The “true” residual displacement of SCVEB was 5.77 − 4.88 = 0.89 mm, which was an accepted level. 

Figure 12d,e gives the calculated *E*_D_ and *EVD* value of the specimens. It can be seen that the energy-dissipation capacity increases with increasing loading amplitudes; however, the *EVD* shows the opposite trend. This is because the *EVD* is only related to the shape of the hysteretic curve, where a greater amount of increase is accumulated for *E*_S_ rather than *E*_D_, and, hence, it decreases the *EVD*. The *EVD* indicator also reflects that the larger the deformation, the less plump the hysteresis loop is. Thanks to the extra energy dissipation source provided by the four VEDs, both energy-dissipation indicators (*E*_D_ and *EVD*) show that the SCVEB has better energy-dissipation capacity than the SCB, and such superiority is more evident under large loading amplitudes. It seems that the SCVEB exhibited a “better” energy-dissipation performance under low loading frequency. However, it should be noticed that the 1.0 Hz-series comes after the 0.1 Hz series in this experiment. Fatigue damage may be accumulated in the rubber layer as the experiment progresses, and, thus, the energy-dissipation property of ED system may decrease to some extent. In fact, the loading frequency may have negligible influence on the energy-dissipation performance of the SCVEB in this test, since the rubber layer has been proven to be rate-unsensitive in Section 3.2. 

A component-level simulation work was conducted to verify the effectiveness of the selected hysteretic models that is further to be adopted in the following-up system-level analysis. A modified SelfCentering model (i.e., the four-segment simplified model mentioned in Section 2.1) was used to capture the hysteresis behavior of the SMA cables and the values calibrated in Section 3.1 was adopted. The hysteresis behavior of VEDs was simulated by BoucWen Material model in OpenSEES. Moreover, the ElasticMultiLinear Material model was used to consider the decompression process of the pre-tensioned SMA cables and the MultiLinear Material model was adopted to consider the extra friction between elements in SCVEB. The simulation results are plotted in Figure 12f. It can be seen that the simulation curves fit well with the tested results, proving the validity of this modeling method.

## 5. System-Level Analysis

### 5.1. Prototype Buildings

**Figure 13:**
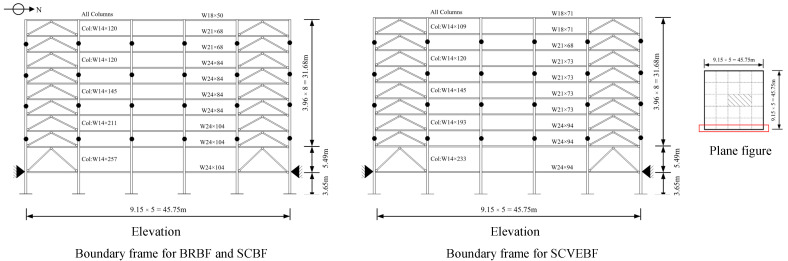
Nine-story prototype building.

For an in-depth understanding of the fundamental performance of structural systems employing the proposed SCVEB, a system-level analysis was conducted. Three nine-story steel frames employing different types of braces (i.e., SCVEB, SCB, and conventional buckling restrained brace) were designed and analyzed for comparison. All of these frames employ the concentrically inverted-V-type braces with the same arrangements. These frames were designed according to ASCE 7-16 [85] by the modal-response spectrum analysis method. Figure 13 shows the basic information of the frames.

These frames were assumed to be located at a stiff soil site (Site Class D) in Los Angeles. Importance factor *I*_e_ = 1.0, response modification coefficient *R* = 8.0, deflection amplification factor *C*_d_ = 5.0, design response spectral values S_DS_ = 2/3S_MS_ = 1.376 g, and S_D1_ = 2/3S_M1_ = 0.707 g, are considered.

### 5.2. Design and Modeling

For the nonlinear dynamic analysis in OpenSEES, centerline models which represent half of the buildings in the North–South (NS) direction were established (see Figure 13). The basic information of these frames is provided in Table 1. The beam-to-column connections are assumed to be rigid for all of these three buildings to maintain certain redundancy against earthquake [77]. When modeling, the boundary frame members are simulated by the Steel01 material with idealized kinematic hardening. The detailed modeling information of these frames are described in the following sections.

#### 5.2.1. Conventional Buckling Restrained Brace Frame (BRBF)

The BRBs were modeled with “truss” elements, and the Steel02 Giuffre–Menegotto–Pinto material model was used. The seismic weight was appropriately distributed to the main frame and the adjacent lean columns. The Rayleigh damping ratio was adopted as 5% for the first and third modes of vibration.

#### 5.2.2. Typical Self-Centering SMA Cable-Based Frame (SCBF)

The information of the employed SMA cables is provided in Table 1. The effective length of the SMA cables was determined by ensuring that the strain not exceeding 10% at an inter-story drift is around 5%. The preload was determined by the “yield” force of the SMA cables (see Figure 6). The SCBs were modeled with “truss” elements, and a modified SelfCentering material model was used to capture the unique flag-shaped hysteresis behavior. An energy dissipation factor (*β*) of 0.6 was assumed for SMA cables [53]. 

It is worth noting that the SCBF and BRBF are designed to have the same initial stiffness and boundary frame. This is because experiment results have revealed that the actually measured initial stiffness of SCB specimens is comparable to those of the BRBs with a similar level of load-carrying capacity [18]. Through this design, the main difference between these two frames lies in the energy-dissipation capability, and the energy dissipation provided by the two types of braces can be compared more intuitively.

#### 5.2.3. Frame with SCVEB (SCVEBF)

When modeling the SCVEBF, a modified boundary frame with an approximately 25% reduction in the overall strength of the structure is considered. This is because ASCE 7–16 [85] allows for a 25% reduction in the base shear for damped structures, with the perspective of saving the overall cost of construction. The basic information of the prototype SCVEBF is shown in Figure 13 and summarized in Table 1. The first-modal-added damping ratio, *ξ*_add_, [85] of structure SCVEBF provided by the viscoelastic material is assumed to be around 0.1. A total of eight viscoelastic material layers are considered for each brace, and the geometric information of each layer is summarized in Table 1. The VEDs were simulated by using the Kelvin–Voigt model in OpenSEES, which involves paralleled viscous and elastic spring elements [63], and the associated parameters for simulating viscoelastic material properties are chosen from the work performed by Zimmer [86] rather than the test results in this paper. It is noteworthy that the models proposed in References [87,88] can be employed to simulate the nonlinear viscoelastic hysteretic behavior. However, since the linear viscoelastic materials are much more mature than the nonlinear ones and have been adopted by more independent researchers, for ease of comparison with previous existing results, the follow-up system-level analysis is conducted based on the linear viscoelastic hysteretic behavior.

### 5.3. Structural Performance

**Figure 14:**
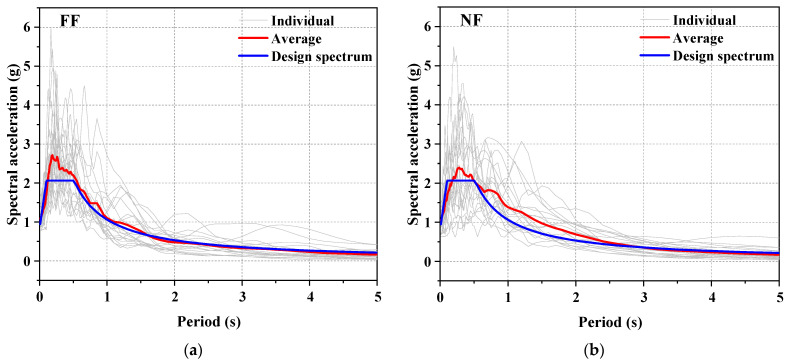
Response spectra of selected ground motions: (**a**) far-field (FF) ground motion records and (**b**) pulse-like near-fault (NF) ground motion records.

A nonlinear time-history analysis was carried out to examine their seismic performance. A total of 20 far-field (FF) and 20 pulse-like near-fault (NF) ground motions at the MCE level were considered herein. The FF records were selected from the FEMA P695 database [89] and were scaled to fit the target spectrum (see Figure 14a). As for NF records, scaling should be cautiously performed for NF ground motions, since some key pulsing characteristics may be violated by uniform scaling [90]. In this paper, the 20 NF ground motions were carefully selected according to the criteria proposed by Baker [91] to match the design spectrum (see Figure 14b). It is confirmed that the mean spectrum of the NF records is not less than the design response spectrum for periods ranging from 0.2*T*_1_ to 2.0*T*_1_, which satisfies the ASCE 7–16 requirements.

#### 5.3.1. Peak Inter-Story Drift (PID)

**Figure 15:**
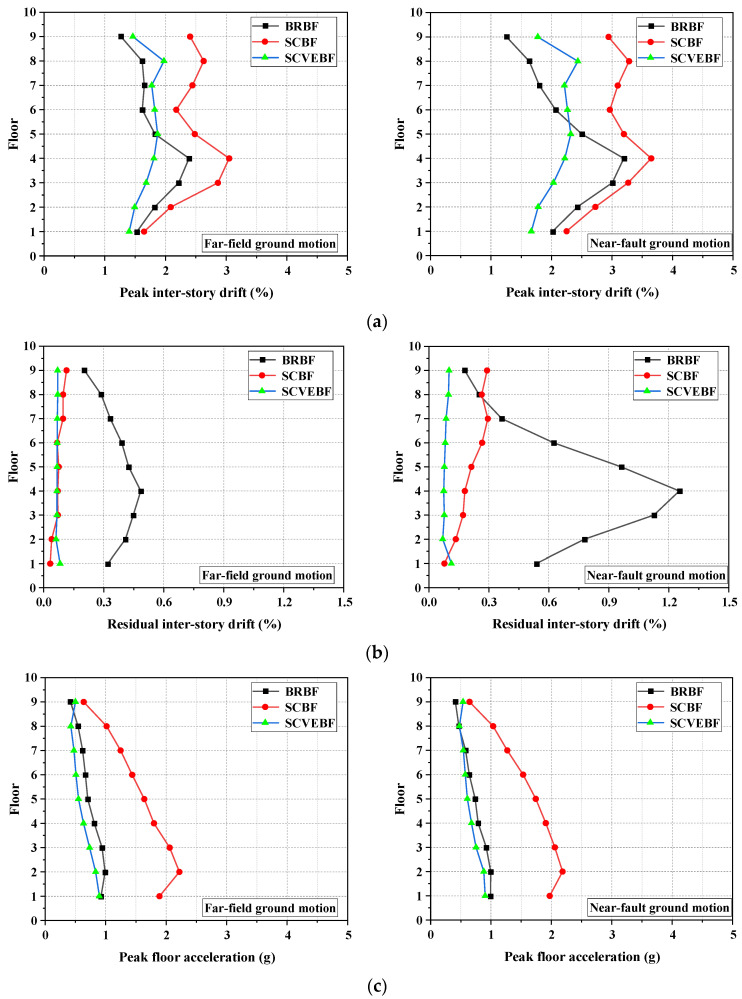
Mean responses of structures: (**a**) peak inter-story drift (PID), (**b**) residual inter-story drift (RID), and (**c**) peak absolute floor acceleration (PFA).

The PIDs of the frames are shown in Figure 15a. The results show that the PIDs of the SCVEBF are all smaller than that of SCBF, with the maximum PID of the SCVEBF being reduced by 35% and 33% compared to SCBF under FF and NF ground motions, respectively. It demonstrates that the use of the viscoelastic material is highly effective in controlling the PID. Extra energy-dissipation capacity provided by the viscoelastic material is responsible for this improvement. Moreover, the maximum PID of the SCVEBF is even less than BRBF; this, again, confirms the feasibility of the proposed SCVEBF.

#### 5.3.2. Residual Inter-Story Drift (RID)

The RIDs of the frames are shown in Figure 15b. Both the SCBF and SCVEBF exhibit reduced residual deformation compared to the BRBF, thus confirming the effectiveness in RID control for the SCBF/SCVEBF. Due to the pulsing effect, the RIDs of the SCBF increased under the NF earthquakes compared with those under FF earthquakes, where the increased residual deformation of SCBF is mainly attributed to the inelastic deformation of the boundary frame. By contrast, the RIDs of the SCVEBF at each story remain basically the same, and there is almost no difference observed between the results under FF and NF earthquakes. It is believed that the added damping ratio contributed by the viscoelastic material suppresses the RID especially for the upper floors. These results, again, prove that the proposed SCVEBF is a promising solution for controlling residual drifts that is especially effective in near-fault region.

#### 5.3.3. Absolute Peak Floor Acceleration (PFA)

The height-wise peak floor acceleration (PFA) responses of the structures are shown in Figure 15c. It is found that the SCBF exhibits the maximum PFA. This is because the unique flag-shaped hysteresis loop of SCBF that features an abrupt “transition points” under reverse-loading would induce a large difference in the shear force between the adjacent stories. Relevant research works have reported that inconsistent inter-story shear forces of the two adjacent floors may amplify PFA [92]. However, it is of interest to find that the PFAs of the SCVEBF are significantly smaller than that of the SCBF, and even lower than the BRBF. This is mainly attributed to the participation of viscoelastic material, which can effectively neutralize the sharp “transition points” during unloading [61].

## 6. Conclusions

A novel type of self-centering brace, namely the self-centering SMA-viscoelastic hybrid brace (SCVEB), was proposed in this study. The energy dissipation was provided by the SMA cables, as well as the viscoelastic dampers (VEDs), whilst the self-centering capacity was provided by the former. The fundamental mechanical behavior of individual SMA cables and viscoelastic dampers was first investigated, followed by a more comprehensive experimental study on a proof-of-concept SCVEB specimen. The main findings and conclusions are summarized as follows:

(1) The SMA cable exhibits typical flag-shaped hysteretic loops with a large recovery strain. Reasonable cyclic pre-training is suggested before anchoring to SCVEB, since this process was shown to help stabilize the hysteretic response.

(2) The VED is capable of providing reliable energy dissipation. The rubber in the test did not show rate-dependence property, such as typical viscoelastic material, and this may be due to the differences in their compositions.

(3) The SCVEB specimen exhibited satisfactory self-centering and energy-dissipation capability, although a certain degree of residual strain was observed due to manufacturing error. The participation of viscoelastic material, indeed, enhanced the energy-dissipation capability. 

(4) The system-level analysis shows that the frames employing the proposed SCVEB have satisfied peak inter-story drifts under the MCE and almost negligible residual inter-story drifts. More importantly, the SCVEB can further reduce the peak floor acceleration of the frames. These encouraging findings demonstrate that the proposed SCVEB could be a cost-effective self-centering solution by reducing member size of the boundary frame with less SMA consumption.

## Figures and Tables

**Table 1:** Basic information of frames.

	BRBF			SCBF			SCVEBF			
Story	*k*_ini_ (kN/mm)	*F*_y_ (kN)	*A* (mm^2^)	No.	*l*_SMA_ (mm)	*F*_p_ (kN)	No.	*l*_SMA_ (mm)	*F*_p_ (kN)	*L* × *W × H* (mm × mm)
1	400.4	2448.2	10,418	32	1760	2407	20	1760	1504	500 × 280 × 30
2	329.6	1706.3	7261	22	1500	1655	15	1500	1128	500 × 210 × 30
3	256.2	1326.1	5643	18	1500	1354	13	1500	978	500 × 180 × 30
4	228.9	1185.1	5043	16	1500	1203	12	1500	902	500 × 160 × 30
5	223.6	1157.4	4925	16	1500	1203	10	1500	752	500 × 140 × 30
6	185.1	958.1	4077	12	1500	902	9	1500	677	500 × 125 × 30
7	155.4	804.6	3424	10	1500	752	8	1500	602	250 × 210 × 30
8	102.0	528.3	2248	7	1500	526	4	1500	301	250 × 110 × 30
9	61.9	320.3	1363	4	1500	301	2	1500	150	250 × 70 × 30

Note: “*k*_ini_” refers to initial stiffness of BRB, “*F*_y_” refers to yield force of BRB, “*A*” refers to the area of BRB’s steel core, “No.” refers to the number of SMA cables, “*l*_SMA_” refers to the length of SMA cables, “*F*_p_” refers to the total preload of SMA cables, and “*L* × *W*× *H”* refers to the geometric dimension of the rubber layer.

## Data Availability

Data presented in this study are available in this article.
